# Priming European Sea Bass Female Broodstock Improves the Antimicrobial Immunity of Their Offspring

**DOI:** 10.3390/ani13030415

**Published:** 2023-01-26

**Authors:** Yulema Valero, Luis Mercado, Marta Arizcun, Alberto Cuesta, Elena Chaves-Pozo

**Affiliations:** 1 Oceanographic Centre of Murcia, Spanish Institute of Oceanography, Spanish National Research Council, Carretera de la Azohía s/n, Puerto de Mazarrón, 30860 Murcia, Spain; 2 Grupo de Marcadores Inmunológicos, Laboratorio de Genética e Inmunología Molecular, Instituto de Biología, Pontificia Universidad Católica de Valparaíso, Valparaíso 2362807, Chile; 3 Immunobiology for Aquaculture Group, Department of Cell Biology and Histology, Faculty of Biology, Regional Campus of International Excellence “Campus Mare Nostrum”, University of Murcia, 30100 Murcia, Spain

**Keywords:** broodstock, protein-expressing vector, antimicrobial peptides, antimicrobial function, innate immune response, *Dicentrarchus labrax*

## Abstract

**Simple Summary:**

Immunity is a key factor in the development of fish embryos, as they are exposed to multiple pathogens. In vertebrates, the maternal transfer of immunity influences the acquisition of immunocompetence. In this work, we demonstrate that in fish, broodstock female priming close to the spawning season improves the development of the innate immune system of their progeny. This occurs via the enhancement of several innate activities and the upregulation of the transcriptional levels of different antimicrobial peptides. Moreover, we provide evidence of the existence of the maternal transfer of immune proteins related to antimicrobial responses in European sea bass. This work also demonstrates that the use of a CP-NNV-expressing vector for priming females is safe, as the plasmid was not detected in their progeny.

**Abstract:**

Acquiring immunocompetence is essential in the development of fish embryos, as they are exposed to environmental pathogens even before they are fertilized. Despite the importance of the antimicrobial function as the first line of defense against foreign microorganisms, little knowledge is available about its role in larval development. In vertebrates, transgenerational immune priming influences the acquisition of immunocompetence of specimens, regulating the selective allocation of nongenetic resources to their progeny and modulating their development. In this work, we primed teleost European sea bass broodstock females with a viral protein expression vector in order to evaluate the innate immunity development of their offspring. Several antimicrobial functions, the pattern of expression of gene coding for different antimicrobial peptides (AMPs), and their protein levels, were evaluated in eggs and larvae during development. Our data determined the presence of antimicrobial proteins of maternal origin in eggs, and that female vaccination increases antimicrobial activities and the transcription and synthesis of AMPs during larval development.

## 1. Introduction

Fish eggs and larvae in early stages of development are in close contact with all aquatic pathogens, even when their immune system is not yet fully developed [1]. Fish immunocompetence is acquired during the early stages of larval development, although the moment when it becomes fully functional depends on many factors, including environment (mainly temperature), age, and species [2]. In European sea bass (*Dicentrarchus labrax*), an important fish species in the Mediterranean, complete immunological maturity is achieved between 137- and 145-days post-hatching (dph), when adult levels of T and B lymphocytes are reached [3]. Before then, fish immunity is based on innate responses. Thus, lysozyme activity has been detected for the first time in newly fertilized sea bass eggs [4] as well as other molecules such as cathepsins. The latter may also play a bactericidal role and are detectable from morula stages (12–16 cells) at about 3.2 h post-fertilization (hpf) in several species [4,5,6,7]. As sea bass larvae completely depend on innate immunity during the first stages of larval development, the study of how to improve their innate immunity seems worthwhile, given the great economic importance of this species for the Mediterranean aquaculture sector.

To date, the main focus of broodstock immunization studies on both fish and mammals has been on the protection of their progeny through the improvement in adaptive immunity, using the titration of specific antibodies as a marker of vaccine effectiveness [8]. Therefore, the maternal transfer of specific factors against a concrete pathogen after broodstock immunization has been noted [1]. However, only few works have described the presence of antimicrobial activities such as lysozyme in eggs and larvae in the early stages of development beyond the maternally transferred antibodies [9,10,11].

Immunological memory is considered a specific characteristic of adaptive immunity. However, during the last decade, relevant investigations have documented trained immunity, namely, the capacity of innate immunity to orchestrate improved infection resistance upon second challenges in mice and humans [12,13]. In intensive aquaculture, different commonly used compounds induce the activation of innate mechanisms and the production of non-specific factors that, later on, improve resistance to pathogens [14]. Several studies on invertebrates and vertebrates show that maternal priming, apart from the direct transfer of adaptive immunity, could result in the inheritance of trained innate immunity, which may be critical in the immune response of progeny [15,16,17,18,19]. Additionally, in fish, transgenerational immune priming has been observed beyond F1 generation [20]. Thus, the transfer of trained innate immunity in fish offers an interesting and attractive approach to improve the innate immune response in progeny in the early stages of development, when specific immunity has not yet been acquired.

Multiple studies have demonstrated the relevance of the antimicrobial function in the fight against pathogens [21]. Hepcidin, Dicentracin, and Nk-lysin are potent AMPs that have antimicrobial functions as well as immune regulatory roles, and which expression is modulated upon bacterial, viral, or parasitic infections in European sea bass [22,23,24,25]. Indeed, Nk-lysin- and Dicentracin-derived peptides in European sea bass have been demonstrated to exert robust antibacterial and antiviral activities [26]. Thus, we used a DNA vaccine expressing the capsid protein of nodavirus (CP-pNNV), which has been demonstrated to trigger innate immune activity and protection [27], to prime a female broodstock of European sea bass. Subsequently, we evaluated different innate immune functions, the presence of Hepcidin, Nk-lysin, and Dicentracin, as well as the pattern of expression of several AMP coding genes in the eggs and larvae of their F1 progeny. We demonstrate that antimicrobial factors are transferred from females to eggs, and that the priming of females enhances innate immunity in their progeny, resulting in a viable solution to consumer acceptance, as the plasmid is not detected in their offspring.

## 2. Materials and Methods

### 2.1. Animals

Mature specimens of 3-year-old European sea bass (*Dicentrarchus labrax*), with a mean body mass (bm) of 1518 ± 85 g were bred and kept at the facilities of the Oceanographic Centre of Murcia, Spanish Institute of Oceanography (COMU-IEO), CSIC, in Mazarrón (Spain).

### 2.2. Nodavirus-Expressing Vector Constructs

We used a DNA vaccine (CP-pNNV) by cloning the entire open reading frame of the NNV capsid protein gene into the expression vector pcDNA3.1/V5-His-TOPO [27]. A relegated empty pcDNA3.1/V5-His-TOPO plasmid (pcDNA3.1) was used as negative control.

### 2.3. Experimental Design and Sampling Procedure

Healthy European sea bass broodstocks were anesthetized with clove oil (20 ppm), and males and females identified after gentle abdominal massage. Forty healthy adult fish were divided into two groups composed of 5 females and 15 males each, which were kept in separate 15 m^3^ round tanks of natural seawater (38‰ salinity), temperature (around 13 C), and photoperiod (Latitude 37.5667, Longitude −1.2; January–February). A subcutaneous magnetic tag code was implanted in each female, which was primed by intramuscular injection with 50 µL of 1 µg/µL of CP-pNNV in 50 mM phosphate-buffered saline pH 7.2 (PBS) or with 1 µg/µL of pcDNA3.1 in PBS as a mock-primed fish (control). The specimens were vaccinated twice within 15 days in order to ensure optimal priming of their immune system.

The first spawn from each group occurred spontaneously at day 20 of the experiment (5 days after the last priming), the moment at which samples of eggs were harvested and the larvae bred in 5000 L round tanks, with an initial density of 100 larvae/L. The temperature was 13 C at harvesting, increased naturally to 19 C at the end of the experiment. The fry were kept in the dark until the day at which the sum of each day temperature reached 160 °C, and subsequently, with 16 h light:8 h dark photoperiod and a light intensity of 500 lux at the water surface. Larvae were fed with *Artemia* nauplii (Inve Animal Health, Vigo, Spain) until 18 days post-hatching; with enriched Instar II *Artemia* until 45 days post-hatching; and subsequently, with a commercial dry pellet diet (Skretting, Stavanger, Norway). Three pools of eggs (500 mg each) were sampled at 0 and 2 days post-fertilization (dpf), while three pools of larvae samples (300 mg each) were sampled at 5, 7, 9, 12, 15, 19, 23, 26, 40, and 69 dpf, and stored in TRIzol^®^ Reagent (Invitrogen, Waltham, MA, USA) at −80 °C for later gene expression analysis. Another three pools of eggs and larvae were homogenized in 1 mL of PBS. Eggs were sonicated on ice in 30 s cycles until complete homogenization, while larvae were mechanically homogenized. Homogenates were centrifuged at 10,000× *g* for 10 min at 4 C and the supernatants collected and stored at −80 °C for later protein quantification and functional analysis.

### 2.4. Functional Determination of Immunity

In all samples, the protein concentration was determined according to Bradford’s method using bovine serum albumin (BSA, Sigma, St. Louis, MO, USA) as the standard [28]. Peroxidase, protease, anti-protease, lysozyme, and total bactericidal activities were analyzed as follows.

Peroxidase activity was measured according to a previous protocol [29] and modified as described elsewhere [30]. Wells with Hank’s balanced salt solution (HBSS) but no sample were used as blanks. Samples were run in triplicate. One unit was defined as the amount of protein producing an absorbance change of 1 and the activity was expressed as U/mg of egg or larvae homogenate.

The protease activity was determined as the percentage of hydrolysis of azocasein using a previous protocol [31] and modified as described elsewhere [30]. For a positive control, 10 µL of 2 mg/mL proteinase K (AppliChem, Darmstadt, Germany) in PBS replaced the sample (100% of activity); for a negative control, PBS replaced the sample (0% of activity). Samples were run in duplicate. The percentage of protease activity for each sample was calculated as the % of the activity of the positive control. Results were expressed as % of activity/mg of egg or larvae homogenate.

Anti-protease activity was determined by the ability of the samples to inhibit proteinase K activity using a previous protocol [32] and modified as described elsewhere [30]. For a positive control (100% of anti-protease activity), PBS replaced samples and proteinase K; for a negative control (0% of anti-protease activity), PBS replaced only the sample. Samples were run in duplicate. The percentage of inhibition of proteinase K activity for each sample was calculated as 100−(% of sample activity). Results were expressed as a % of activity/mg of egg or larvae homogenate.

Lysozyme activity was measured using a modified turbidimetric method [33] as described elsewhere [34]. One unit of lysozyme activity was defined as a reduction in absorbance of 0.001/min. A standard curve from 20 µg/mL to 0.3 µg/mL of hen egg white lysozyme (HEWL, Sigma) and a blank control (negative control) were established. Standards and samples were run in duplicate, and all the measures were corrected with the blank. The results were expressed as U/mg of egg or larvae homogenate.

The bactericidal activity of samples was determined by evaluating their effect on the bacterial growth of *Vibrio harveyi* (Vh) (strain Lg 16/100) curves using a modified, previously described method [35] and briefly described elsewhere [30]. Samples replacing bacteria by culture medium were used as blanks (negative control). Samples replacing serum or homogenates by culture medium were used as positive controls (100% growth or 0% antibacterial activity). Samples and controls were run in duplicate. Bactericidal activity was expressed as 100−(% of bacterial growth). Results were corrected, with the absorbance measured in each sample at the initial time point and expressed as % of activity/mg of egg or larvae homogenate.

### 2.5. Gene Expression Analysis

Total RNA was isolated from samples frozen in TRIzol Reagent^®^ (Invitrogen) following the manufacturer’s instructions. A total of 1 µg of total RNA was treated with DNAse I (1 unit/µg RNA, Promega, Madison, WI, USA) to remove genomic DNA. The first strand of cDNA was synthesized by reverse transcription using the Superscript III (Invitrogen) with an oligodT12-18 primer, followed by RNAse H (Invitrogen) treatment for 60 min at 50 °C. The expression of the genes codifying for the AMPs, Hepcidin (*hamp*), complement factor 3-1 and 3-2 (*c3*), lysozyme (*lyz*), and Nk-lysin (*nk-lys*),was analyzed in real-time PCR, using an ABI PRISM 7500 instrument (Applied Biosystems) and SYBR Green PCR Core Reagents (Applied Biosystems), as previously described [36]. Reaction mixtures were incubated for 10 min at 95 °C, followed by 40 cycles of 15 s at 95 °C, 1 min at 60 °C, and finally, 15 s at 95 °C, 1 min at 60 °C, and 15 s at 95 °C. The specific primers were designed using the Oligo Perfect software tool (Invitrogen) and shown in Table 1. Before the experiments, the specificity of each primer pair was studied using positive and negative samples. A melting curve analysis of the amplified products validated the primers for specificity. Negative controls with no template were always included in the reactions. For each mRNA sample, gene expression was corrected by the geometric average of the endogenous elongation factor 1 alpha (*ef1a*) and ribosomal protein L13 alpha (*l13a*) coding gene content in each sample. This was, expressed as 2^−ΔCt^, where ΔCt is determined by subtracting the endogenous Ct geometric average value from the target Ct. The reference genes, *ef1a* and *l13a*, were chosen based on the stability of their Ct values and the stability of their pattern of expression.

In addition, a convectional PCR was performed using F2 and R3 primers (Table 1) [37], which are specific for the capsid NNV gene and able to detect the vaccine. Products were run in 1.5% agarose gel for visualization.

### 2.6. Peptide Quantification by ELISA

Protein detection was performed by an indirect ELISA as elsewhere [38]. Thus, 10 µg of total protein from eggs and larvae homogenates was incubated overnight at 4 °C in 96 MaxiSorp flat-bottomed plates (Nunc, Roskilde, Denmark), and then washed with PBS containing 0.2% Tween-20 (PBST). Samples were blocked with 5% skimmed milk in PBST for 1 h at RT and incubated with the optimal dilution (1:100) of the primary antiserum (mouse immune sera against rainbow trout Hepcidin or against European sea bass Nk-lysin or Dicentracin [24]) for 1 h at RT. After three washes of 5 min with PBST, the anti-mouse IgG-HRP (Thermo Fisher Scientific, Waltham, MA, USA) at the optimal dilution of 1:7000 was incubated for 1 h at RT. The reaction was detected by incubating 100 µL per well of TMB single solution (Invitrogen) for 20 min at RT and stopped with 50 µL of sulfuric acid 1 N and read at 450 nm usinga VERSA max microplate reader (Molecular Devices, San Jose, CA, USA). All assays were performed in duplicate. Positive controls were used, consisting of sample substitution by Nk-lysin, Hepcidin, or Dicentracin synthetic epitope peptides. Negative controls with no sample or primary antiserum were always included.

### 2.7. Nomenclature and Statistical Analysis

The genetic nomenclature used in this manuscript follows the guidelines of the Zebrafish Nomenclature Committee (ZNC) for fish genes and proteins and the HUGO Gene Nomenclature Committee for mammalian genes and proteins.

The BestKeeper algorithm index and repeated pair-wise regression analysis was applied to determine the stability of the housekeeping genes’ Ct values throughout the experiment. BestKeeper^®^ software was used according to the methodology previously described in [39]. The data were analyzed using Student’s *t*-test to establish differences between control and vaccinated groups at each time point (*p* ≤ 0.05). Data are represented as the mean ± standard error of the mean (SEM).

## 3. Results

Viral mRNA was detected neither in the eggs nor in the larvae from primed females (data not shown).

### 3.1. Antimicrobial Activities Are Enhanced in Larvae from Vaccinated Females

**Figure 1:**
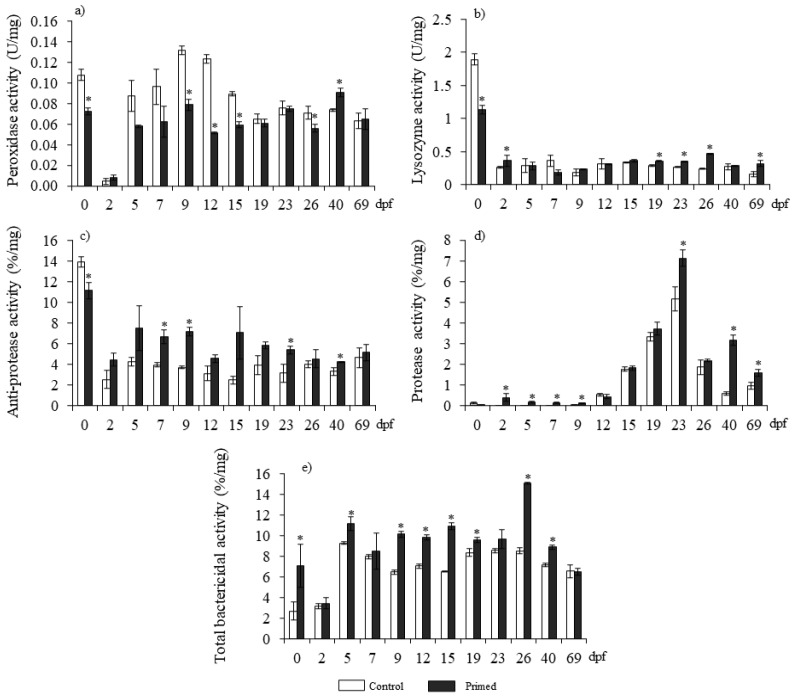
Peroxidase (**a**), lysozyme (**b**), anti-protease (**c**), protease (**d**) and, total bactericidal (**e**) activity in eggs (0 or 2 dpf) and larvae (from 5 to 69 dpf) homogenates from control or primed females’ progeny. Data represent the mean ± SEM (*n* = 3). Asterisks denote statistical differences with controls (*p* ≤ 0.05). dpf denotes days post-fertilization.

Although peroxidase, lysozyme, and anti-protease activities were inhibited at some early time points of larval development in the primed group compared with the control, all the activities were upregulated later on during larval ontogeny (Figure 1). Thus, in fry from primed females, peroxidase activity decreased at 0, 9, 12, 15, and 26 dpf, and increased after 40 dpf (Figure 1a). Lysozyme and anti-protease activities were decreased at 0 dpf, and increased at 2, 19, 23, 26, and 69 dpf or 7, 9, 23, and 40 dpf, respectively (Figure 1b,c). However, protease and bactericidal activities were increased in the fry from primed females compared with the control fry. Thus, protease activity was stimulated at 2, 5, 7, 9, 23, 40, and 69 dpf (Figure 1d), irrespective of whether bactericidal activity was at 0, 5, 9, 12, 15, 19, 26, or 40 dpf (Figure 1e).

### 3.2. AMPs Transcription Was Upregulated in the Progeny of CP-pNNV-Primed Females

**Figure 2:**
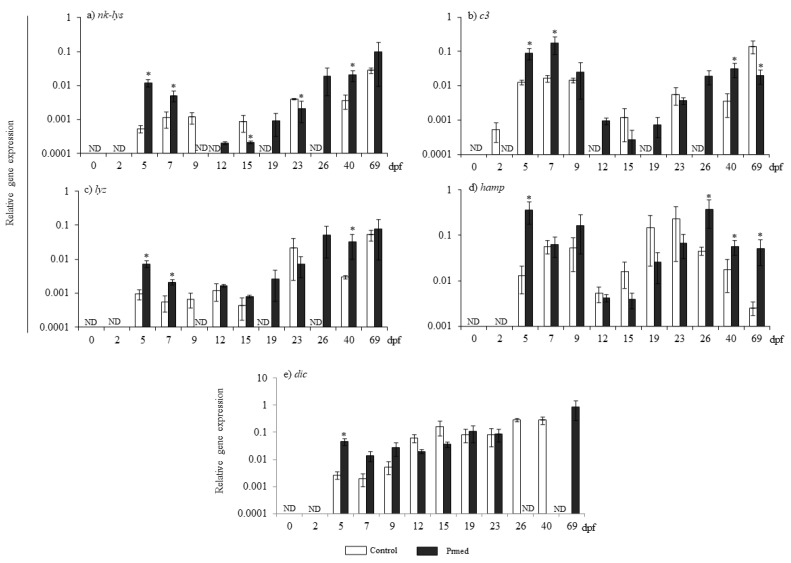
Transcript levels of gene coding for Nk-lysin (*nk-lys*) (**a**), complement factor 3-1 and 3-2 (*c3*) (**b**), lysozyme (*lyz*) (**c**), Hepcidin (*hamp*) (**d**), and Dicentracin (*dic*) (**e**) in eggs (0 or 2 dpf) and larvae (5 to 69 dpf) homogenates from control or primed females’ progeny. Data represent the mean ± SEM (*n* = 3). Asterisks denote statistical differences with controls (*p* ≤ 0.05). dpf denotes days post-fertilization.

No transcript of the genes analyzed was detected in eggs at 0 and 2 dpf from either the control or the primed group (Figure 2) except for *c3* transcripts, which were detected from 2 dpf onwards in the control group (Figure 2b). Thus, in the control group, stable levels of expression were observed in all genes from 5 to 9 dpf. This was followed by a period with low or undetected levels of expression at 12 and 15 dpf in the case of *hamp*, and from 12 to 26 dpf in the case of *nk-lys*, *c3*, and *lyz* (Figure 2a–c), whilst a progressive upregulation of gene transcript levels was found afterwards in all coding genes but *dic* (Figure 2). Exceptionally, *dic* transcriptional levels progressively increased from the beginning to the end of the sampled ontogenetic period (Figure 2e). In all genes, the pattern of expression in the primed fry was similar to the control, although the expression levels were higher in fry from primed females than in fry from control females at certain points (*nk-lys* at 5, 7, 12, 19, 26, and 40 dpf, *c3* at 5, 7, 12, 19, 26, and 40 dpf, *lyz* at 5, 7, 19, 26, and 40 dpf, *hamp* at 5 and 26 onwards dpf, and *dic* at 5 and 69 dpf). However, fry from the primed group also showed punctual downregulations of *nk-lys* and *c3* expression at 15, 23 and 69 dpf, respectively, or a complete blockage of *nk-lys*, *c3*, *lyz*, and *dic* expression (*c3* at 2 dpf, *lyz* and *nk-lys* at 9 dpf, and *dic* at 26 and 40 dpf).

### 3.3. Priming Broodstock Females Enhances Nk-Lysin Levels during Fry Development

**Figure 3:**
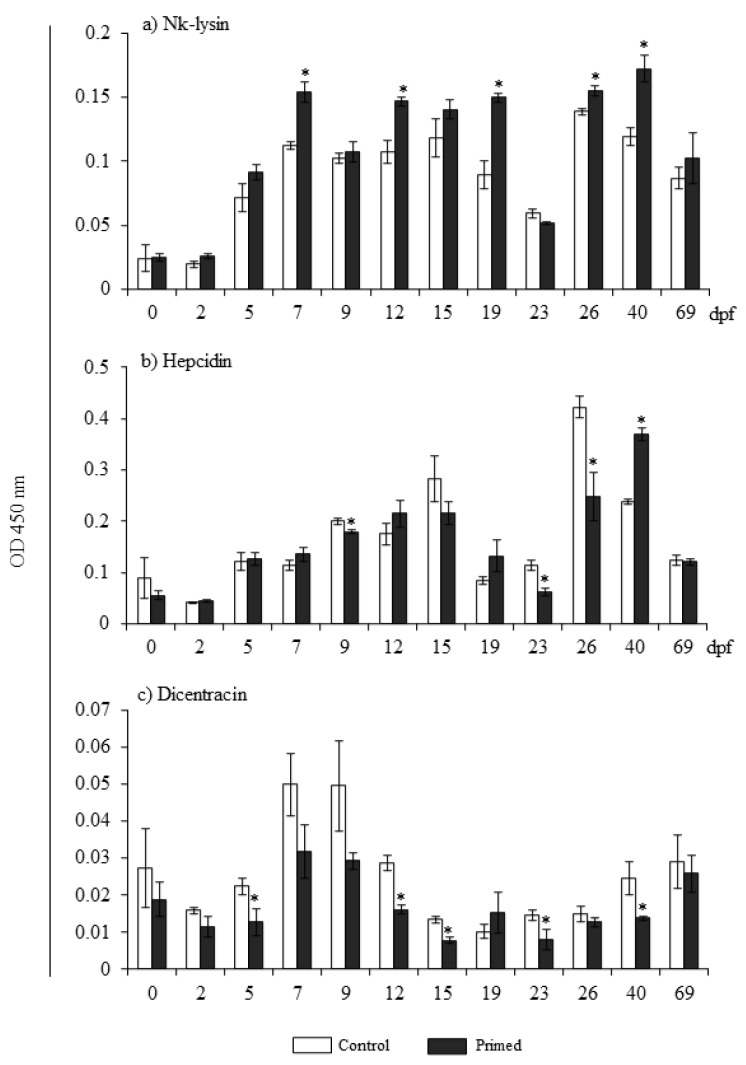
Protein levels of Nk-lysin (**a**), Hepcidin (**b**), and Dicentracin (**c**) in eggs (0 or 2 dpf) and larvae (5 to 69 dpf) homogenates from control or primed females’ progeny. Data represent the mean ± SEM (*n* = 3). Asterisks denote statistical differences with controls (*p* ≤ 0.05). dpf denotes days post-fertilization.

Peptide levels of Nk-lysin, Hepcidin, and Dicentracin were studied using the ELISA methodology (Figure 3). Interestingly, Nk-lysin levels were significantly higher in fry from the primed females than in the controls at 7, 12, 19, 26, and 40 dpf (Figure 3a). However, the levels of Hepcidin and Dicentracin were lower in the primed fry than in the control fry. Regarding Hepcidin, fry from the primed group showed lower levels at several time points until 26 dpf, and higher levels at 40 dpf. However, Dicentracin protein levels showed no increase in the fry from primed females at any time point compared with the control fry (Figure 3b,c).

## 4. Discussion

In fish, the presence of proteins with active antimicrobial activity plays a crucial role in the first line of defense during the early life stages, in which fish have not fully developed effective immunocompetence [1,40]. To the best of our knowledge, the study of innate antimicrobial function after broodstock priming has always been dismissed in favor of the study of specific responses. In order to establish whether primed female fish can improve the innate immune inheritance of their fry, a CP-NNV-expressing vector has been used. This study demonstrates that the use of a CP-NNV-expressing vector to prime females is safe, as the plasmid was not detected in their progeny. This is in line with our previous study, in which plasmid expression was not detected in the posterior gut 3 months upon oral intake [27]. These data clearly suggest that the plasmid might be transitorily expressed, and after eliciting immunity, it is eliminated or silenced. Nonetheless DNA-based compounds remain controversial since DNA-treated fish might be considered a product of genetic manipulation by consumers. The use of DNA-based treatments in broodstock fish appears to be a feasible solution to avoid consumer concerns in this regard, as plasmids have been demonstrated not to surpass muscle fibres where injected or integrate into the genome of host cells in several fish species [41,42,43]. With respect to immunity, the CP-NNV-expressing vector induces innate immune responses, such as the type-I interferon pathway, and confers resistance upon experimental challenge, although it does not induce antibodies [27]. Moreover, the NNV capsid protein acts as an apoptosis inducer, a process that sends a danger signal, eliciting a response from the pattern recognition receptors (PPRs) [44,45]. As fish larvae rely on their innate immunity in the first weeks after hatching [2,3], in this study, we have focused on antimicrobial immune responses in the early larval development process.

In the analyzed offspring, all activities were detected in eggs from 0 dpf onwards, in both control and primed groups. Strikingly, only bactericidal activity was enhanced in eggs obtained 0 dpf from primed females compared with controls. Together with small AMPs, lysozyme and the complement protein,*c3*, are closely involved in bactericidal activity [46,47,48], so we studied the pattern of expression of antimicrobial molecules in eggs from both control and primed females. We observed no transcripts of any genes studied in the control or in the primed eggs, with the exception of *c3*, which was detected at 2 dpf in control sea bass eggs. This early production of the *c3* protein also occurred in common carp (*Cyprinus carpio*) eggs, where *c3* transcripts were described for the first time in yolk syncytial layer embryos at 24 hpf [49]. Interestingly, our data showed that, even when there were no transcripts of the studied antimicrobial molecules in the eggs, they showed functional antimicrobial activity at 0 dpf. Moreover, the analyzed AMPs (Hepcidin, Nk-lysin, and Dicentracin) can be found at these early stages at similar levels in eggs from primed and control females. Although our data do not show an increase in the protein levels in primed female eggs, these eggs had a higher level of bactericidal activity than the control ones. Nevertheless, antibacterial activity is performed by many different effectors and coordinated by a wide range of diverse immune response pathways. All these data, taken together, point to the existence of maternal transfer of AMPs to the offspring in fish; however, further work is needed to clarify which of these molecules could be maternally inherited and which molecular pathways are involved. Supporting these data, several reports have described the ability of larvae of other fish species to respond to infections using AMP molecules [50,51].

Taking into account the humoral innate activity levels observed in larvae from 5 to 69 dpf, our data suggest that larvae from primed females reached higher levels of all these activities at several time points, especially bactericidal activity, with higher levels recorded in imprinted larvae from 5 dpf onwards. These data lead us to hypothesize that the innate immune response of larvae can be imprinted by females and that the improved development of their fry might depend on their pathological history. This hypothesis is also supported by the expression levels of *hamp*, *lyz*, *Nk-lysin*, and *dic*, which were not detected at egg stages, but were upregulated at certain sampling points during development, mainly at 5 or 5 and 7 dpf, and then later, at around 40 or 69 dpf. These two peaks in expression observed in most of the immune-related genes, as well as in the AMP levels analyzed in this work, might be related to the two waves of hematopoiesis, a primitive and a definitive wave, previously described in other vertebrates [52].

Regarding protein levels, larvae from primed females showed lower levels of Hepcidin than those from control females at early time points of development. This is contrary to the gene expression of *hamp*, which was upregulated at those time points. However, Hepcidin increased in imprinted larvae at 40 dpf, reaching higher levels than those from controls. In general, the enhanced protein levels of Hepcidin andNk-lysin, as well as the upregulation of *hamp*, *c3*, *lyz*, *nk-lys*, and *dic* transcription levels in the imprinted larvae around 40 and 69 dpf, coincided with the major development of the immune system of European sea bass [3]. This supports the hypothesis that the immune stimulation of the broodstock females is important for immune system development in their progeny.

## 5. Conclusions

In conclusion, our data demonstrate that the priming of broodstock females improves the development of the innate immune system of their progeny. This occurs via the enhancement of several innate activities, such as peroxidase, lysozyme, protease, anti-protease, and bactericidal activities at certain time points of development. Concomitantly with this antimicrobial function, *nk-lys*, *c3*, *lyz*, *hamp*, and *dic* transcriptional levels were upregulated from the early moments of larval development and the Nk-lysin protein levels at all tested time points and Hepcidin at 40 dpf. These results suggest that female immune stimulation close to the spawning season favors the innate immune system status of their progeny. Additionally, the presence of innate immune functions and AMP protein levels without detected transcripts in recently fertilized eggs (0 dpf) point to the existence of maternal transfer of immune proteins related to antimicrobial responses in European sea bass. Thus, further studies are required to clarify the molecular mechanisms that allow for the inheritance of maternally trained immunity to improve the ontogenetic development of the innate immune system of fish. Furthermore, this work demonstrates that the use of the CP-NNV-expressing vector for priming females is safe, as the plasmid wasnot detected in their progeny.

## Figures and Tables

**Table 1:** Gene accession numbers and primer sequences used for gene expression analysis.

Molecule	Gene	Accession Number		Sequence (5′ →3′)
NNV capsid	*cp*	D38636	F2	CGTGTCAGTCATGTGTCGCT
			R3	CGAGTCAACACGGGTGAAGA
Hepcidin	*hamp*	DQ131605	F	CCAGTCACTGAGGTGCAAGA
			R	GCTGTGACGCTTGTGTCTGT
Complement factor 3-1 and 3-2	*c3*	HM563079 HM563078	F	ACCAAAGAACTGGCAACCAC
			R	CTAGCAGTCGGTCAGGGAAC
Lysozyme	*lyz*	FN667957	F	ATTTCCTGGCTGGAACACAG
			R	GAGCTCTGGCAACAACATCA
Nk-lysin	*nk-lys*	KY801205	F	GAAGAAACACCTCGGGGAAT
			R	GCAGGTCCAACATCTCCTTC
Dicentracin	*dic*	AY303949	F	GGCAAGTCCATCCACAAACT
			R	ATATTGCTCCGCTTGCTGAT
Elongation factor 1 alpha	*ef1a*	FM019753	F	CGTTGGCTTCAACATCAAGA
			R	GAAGTTGTCTGCTCCCTTGG
Ribosomal protein L13 alpha	*l13a*	DT044539	F	GCGAAGGCATCAACATCTCC
			R	AGACGCACAATCTTGAGAGCAG

## Data Availability

Data arecontained within the article.

## References

[1] Zhang S., Wang Z., Wang H. (2013). Maternal immunity in fish. Dev. Comp. Immunol..

[2] Mulero I., Garía-Ayala A., Meseguer J., Mulero V. (2007). Maternal transfer of immunity and ontogeny of autologous immune competence of fish: A mini review. Aquaculture.

[3] DosSantos N.M., Romano N., deSousa M., Ellis A.E., Rombout J.H. (2000). Ontogeny of Band T cells in sea bass (*Dicentrarchus labrax*, L.). Fish Shellfish Immunol..

[4] Cecchini S., Terova G., Caricato G., Saroglia M. (2000). Lysozyme activity in embryos and larvae of sea bass (*Dicentrarchus labrax* L.), spawned by brood stocks fed with vitamin C enriched diets. Bull. Eurn. Assoc. Fish. Pathol..

[5] Carnevali O., Mosconi G., Cambi A., Ridolfi S., Zanuy S., Polzonetti-Magni A.M. (2001). Changes of lysosomal enzyme activities in sea bass (*Dicentrarchus labrax*) eggs and developing embryos. Aquaculture.

[6] Cho J.H., Park I.Y., Kim M.S., Kim S.C. (2002). Matrix metalloproteinase 2 is involved in the regulation of the antimicrobial peptide parasin I production in catfish skin mucosa. FEBS Lett..

[7] Cucchi P., Sucré E., Santos R., Leclère J., Charmantier G., Castille R. (2011). Embryonic development of the sea bass *Dicentrarchus labrax*. Helgol. Mar. Res..

[8] Mondal H., Thomas J. (2022). Are view on the recent advances and application of vaccines against fish pathogens in aquaculture. Aquacult. Int..

[9] Hanif A., Bakopoulos V., Dimitriadis G.J. (2004). Maternal transfer of humoral specific and non-specific immune parameters to seabream (*Sparus aurata*) larvae. Fish Shellfish Immunol..

[10] Hanif A., Bakopoulos V., Leonardos I., Dimitriadis G.J. (2005). The effect of seabream (*Sparus aurata*) broodstock and larval vaccination on the susceptibility by *Photobacterium damselae* subsp. *piscicida* and on the humoral immune parameters. Fish Shellfish Immunol..

[11] Nurani F.S., Sukenda S., Nuryati S. (2020). Maternal immunity of tilapia broodstock vaccinated with polyvalent vaccine and resistance of their offspring against *Streptococcus agalactiae*. Aquac. Res..

[12] VanderMeer J.W.M., Joosten L.A.B., Riksen N., Netea M.G. (2015). Trained immunity: A smart way to enhance innate immune defence. Mol. Immunol..

[13] Quintin J., Saeed S., Martens J.H.A., Giamarellos-Bourboulis E.J., Ifrim D.C., Logie C., Jacobs L., Jansen T., Kullberg B.J., Wijmenga C. (2012). *Candida albicans* infection affords protection against reinfection via functional reprogramming of monocytes. Cell Host Microb..

[14] Zhang Z., Chi H., Dalmo R.A. (2019). Trained innate immunity of fish is a viable approach in larval aquaculture. Front. Immunol..

[15] Beemelmanns A., Roth O. (2016). Biparental immune priming in the pipefish *Syngnathus typhle*. Zoology.

[16] Roth O., Joop G., Eggert H., Hilbert J., Daniel J., Schmid-Hempel P., Kurtz J. (2010). Paternally derived immune priming for offspring in the redflour beetle, *Tribolium castaneum*. J. Anim. Ecol..

[17] Hong M., Sandalova E., Low D., Gehring A.J., Fieni S., Amadei B., Urbani S., Chong Y.S., Guccione E., Bertoletti A. (2015). Trained immunity in new born infants of HBV-infected mothers. Nat. Commun..

[18] Norouzitallab P., Baruah K., Biswas P., Vanrompay D., Bossier P. (2016). Probing the phenomenon of trained immunity in invertebrates during a transgenerational study, using brine shrimp *Artemia* as a model system. Sci. Rep..

[19] Green T.J., Helbig K., Speck P., Raftos D.A. (2016). Primedforsuccess:Oyster parents treated with poly(I:C) produce offspring with enhanced protection against Ostreid herpes virus type I infection. Mol. Immunol..

[20] Beemelmanns A., Roth O. (2017). Grand parental immune priming in the pipefish *Syngnathus typhle*. BMC Evol. Biol..

[21] Valero Y., Saraiva-Fraga M., Costas B., Guardiola F.A. (2020). Fish antimicrobial peptides: Beyond the fight against pathogens. Rev. Aquac..

[22] Meloni M., Candusso S., Galeotti M., Volpatti D. (2015). Preliminary study on expression of antimicrobial peptides in European sea bass (*Dicentrarchus labrax*) following in vivo infection with *Vibrio anguillarum*. A time course experiment. Fish Shellfish Immunol..

[23] Valero Y., García-Alcázar A., Esteban M.A., Cuesta A., Chaves-Pozo E. (2015). Antimicrobial response is increased in the testis of European sea bass, but not in gilthead seabream, upon nodavirus infection. Fish Shellfish Immunol..

[24] Valero Y., Arizcun M., Cortés J., Ramírez-Cepeda F., Guzmán F., Mercado L., Esteban M.Á., Chaves-Pozo E., Cuesta A. (2020). NK-lysin, dicentracin and hepcidin antimicrobial peptides in European sea bass. Ontogenetic development and modulation in juveniles by nodavirus. Dev. Comp. Immunol..

[25] Álvarez C.A., Acosta F., Montero D., Guzman F., Torres E., Vega B., Mercado L. (2016). Synthetic hepcidin from fish: Uptake and protection against *Vibrio anguillarum* in sea bass (*Dicentrarchus labrax*). Fish Shellfish Immunol..

[26] León R., Ruiz M., Valero Y., Cárdenas C., Guzmán F., Vila M., Cuesta A. (2020). Exploring small cationic peptides of different origin as potential antimicrobial agents in aquaculture. Fish Shellfish Immunol..

[27] Valero Y., Awad E., Buonocore F., Arizcun M., Esteban M.A., Meseguer J., Chaves-Pozo E., Cuesta A. (2016). An oral chitosan DNA vaccine against nodavirus improves transcription of cell-mediated cytotoxicity and interferon genes in the European sea bass juveniles gut and survival upon infection. Dev. Comp. Immunol..

[28] Bradford M.M. (1976). A rapid and sensitive method for the quantitation of microgram quantities of protein utilizing the principle of protein-dye binding. Anal.Biochem..

[29] Quade M.J., Roth J.A. (1997). A rapid, direct assay to measure degranulation of bovine neutrophil primary granules. Vet. Immunol. Immunopathol..

[30] Valero Y., López-Cánovas A.E., Rodenas M.C., Cabas I., García-Hernández P., Arizcun M., García-Ayala A., Chaves-Pozo E. (2020). Endocrine disrupter chemicals affect the humoral antimicrobial activities of gilthead seabream males even upon the cease of the exposure. Sci. Rep..

[31] Charney J., Tomarelli R.M. (1947). A colorimetric method for the determination of the proteolytic activity of duodenal juice. J. Biol. Chem..

[32] Ellis A.E., Stolen J.S., Fletcher T.C., Anderson D.P., Roberson B.S., vanMuiswinkel W.B. (1990). Serum antiproteases in fish. Techniques in Fish Immunology.

[33] Parry R.M., Chandan R.C., Shahani K.M. (1965). A rapid and sensitive assay of muramidase. Proc. Soc. Exp. Biol. Med..

[34] Valero Y., García-Alcázar A., Esteban M.A., Cuesta A., Chaves-Pozo E. (2014). Seasonal variations of the humoral immune parameters of European sea bass (*Dicentrarchus labrax*L.). Fish Shellfish Immunol..

[35] Sunyer J.O., Tort L. (1995). Natural haemolytic and bactericidal activities of seabream *Sparus aurata* serum are affected by the alternative complement pathway. Vet. Immunol. Immunopathol..

[36] Chaves-Pozo E., Guardiola F.A., Meseguer J., Esteban M.A., Cuesta A. (2012). Nodavirus infection induces a great innate cell-mediated cytotoxic activity in resistant, gilthead seabream, and susceptible, European sea bass, teleost fish. Fish Shellfish Immunol..

[37] Nishizawa T., Mori K., Nakai T., Furusawa I., Muroga K. (1994). Polymerase chain reaction (PCR) amplification of RNA of striped jack nervous necrosis virus (SJNNV). Dis.Aquat. Organ.

[38] Santana P.A., Álvarez C.A., Guzmán F., Mercado L. (2013). Development of a sandwich ELISA for quantifying hepcidinin Rainbow trout. Fish Shellfish Immunol..

[39] Pfaffl M.W. (2001). A new mathematical model for relative quantification in real-time RT-PCR. Nucleic Acids Res..

[40] Swain P., Nayak S.K. (2009). Role of maternally derived immunity in fish. Fish Shellfish Immunol..

[41] Anderon E.D., Mourich D.V., Leong J.A. (1996). Gene expression in rainbow trout (*Oncorhynchus mykiss*) following intramuscular injection of DNA. Mol. Mar. Biol.Biotechnol..

[42] Collins C., Lorenzen N., Collet B. (2019). DNA vaccination for finfish aquaculture. Fish Shellfish Immunol..

[43] Kanellos T., Sylvester I.D., Ambali A.G., Howard C.R., Russell P.H. (1999). The safety and longevity of DNA vaccines for fish. Immunology.

[44] Guo Y.X., Wei T., Dallmann K., Kwang J. (2003). Induction of caspase-dependent apoptosis by betanodaviruses GGNNV and demonstration of protein as an apoptosis inducer. Virology.

[45] Magnadottir B. (2006). Innate immunity of fish (overview). Fish Shellfish Immunol..

[46] Bugla-Plskonska G., Kiersnowski A., Futoma-Koloch B., Doroszkiewicz W. (2008). Cooperation between lysozyme and complementsystem in bactericidal action of human serum–is everything already clear?. Cent. Eur. J. Immunol..

[47] Ogundele M.O. (1998). A novel anti-inflammatory activity of lysozyme: Modulation of serum complement activation. Mediat. Inflamm..

[48] Wardlaw A.C. (1962). The complement-dependent bacteriolytic activity of normal human serum. I. The effect of pH and ionic strength and the role of lysozyme. J. Exp. Med..

[49] Huttenhuis H.B., Grou C., Taverne-Thiele A.J., Taverne N., Rombout J.H. (2006). Carp (*Cyprinus carpio* L.) innate immune factors are present before hatching. Fish Shellfish Immunol..

[50] Chettri J.K., Raida M.K., Kania P.W., Buchmann K. (2012). Differential immune response of rainbow trout (*Oncorhynchus mykiss*) at early developmental stages (larvae and fry) against the bacterial pathogen *Yersinia ruckeri*. Dev. Comp. Immunol..

[51] Santana P.A., Guzmán F., Forero J.C., Luna O.F., Mercado L. (2016). Hepcidin, Cathelicidin-1 and IL-8 as immunological markers of responsiveness in early developmental stages of rainbow trout. Dev. Comp. Immunol..

[52] Katzenback B.A., Katakura F., Belosevic M. (2012). Regulation of teleost macrophage and neutrophil cell development by growth factors and transcription factors. New Advances and Contributions to Fish Biology.

